# Data of expression status of miR- 29a and its putative target mitochondrial apoptosis regulatory gene DRP1 upon miR-15a and miR-214 inhibition

**DOI:** 10.1016/j.dib.2017.12.040

**Published:** 2017-12-20

**Authors:** Muhammad Ishtiaq Jan, Riaz Anwar Khan, Abdul Malik, Tahir Ali, Muhammad Bilal, Long Bo, Abdul Sajid, Naseeb Urehman, Nayyar Waseem, Javed Nawab, Murad Ali, Abdul Majeed, Hamid Ahmad, Sohail Aslam, Sadia Hamera, Aneesa Sultan, Mariam Aneesa, Qamar Javed, Iram Murtaza

**Affiliations:** a Signal Transduction Lab, Department of Bio-Chemistry, Faculty of Biological Sciences, Quaid-i-Azam University Islamabad, Islamabad, Pakistan; b Department of Cardiovascular Surgery, Lady Reading Hospital Peshawar, Pakistan; c Livestock and Dairy Development, Veterinary Research Institute, Peshawar, Pakistan; d Peking Union medical College, Beijing, China; e LUMS, Department of Biology, SBASSE, Lahore, Pakistan; f PINST Department, Preston University, Islamabad, Pakistan

**Keywords:** DRP1, miR-15a, Apoptosis, miRNAs inhibition

## Abstract

Data is about the mitochondrial apoptosis regulatory framework genes PUMA, DRP1 (apoptotic), and ARC (anti-apoptotic) analysis after the employment of their controlling miRNAs inhibitors. The data represents putative conserved targeting of seed regions of miR-15a, miR-29a, and miR-214 with respective target genes PUMA, DRP1, and ARC. Data is of cross interference in expression levels of one miRNA family, miR-29a and its putative target DRP1 upon the inhibitory treatment of other miRNAs 15a and 214.

**Specifications Table**

**Table:** 

**Subject area**	Biology
**More specific subject area**	Signal transduction; gene expression regulation
**Type of data**	Bioinformatics data, Qualitative real time PCR data
**How data was acquired**	Bioinformatics tools were used to assess the miRNA targets; miRNA and RNA expression analysis by qRT PCR
**Data format**	Analyzed
**Experimental factors**	Cardiomyocytes primary cell culturing and transfection with miRNA inhibitors
**Experimental features**	To predict mitochondrial apoptosis regulatory genes regulation by microRNA and respective quantitative expression analysis
**Data source location**	Islamabad, Pakistan, 33° 43′ 0″ N, 73° 4′ 0″ E
**Data accessibility**	The data are available with this article
**Related research article**	Jan et al. [1].

**Value of the data**

• Data comprising mitochondrial apoptosis regulatory proteins may be of value to the scientists working in different disorders including valvular heart diseases, heart failure, kidney diseases, and cancer.
• Data showing microRNAs targeting mitochondrial apoptosis regulatory genes may be of potential value for researchers working in the field of translational research.
• Data presenting microRNAs cross interference phenomenon may open some venues for researchers working on miRNAs regulation and signaling.

## 1 Data

**Fig. 1:**
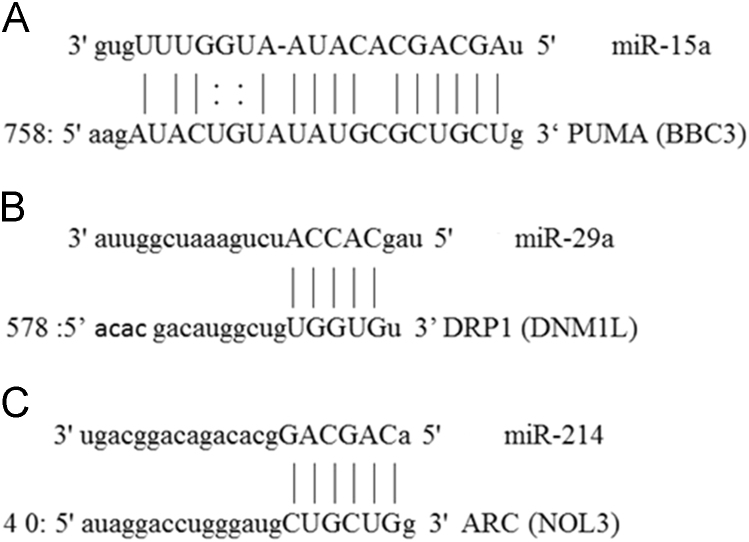
Computational analysis of miRNAs and respective targets (miR-15a, miR-29a and miR-214) and (PUMA, DRP1 and ARC). **A:** miR-15a with complementary region (seed region) in PUMA as its potential putative target. **B:** The sequence and seed region of miR-29a on DRP1. **C:** miR-214 targeted region on ARC gene.

Regulation of Mitochondrial pathology associated genes may be of great merit for multiple disorders with damaged mitochondria as cardiac diseases, cancers and kidney diseases [1]. Present data is about the miRNAs regulation of mitochondrial apoptosis regulatory framework genes PUMA, DRP1 (apoptotic) and ARC (anti-apoptotic). Present data has been generated and confirmed by the multiple miRNAs targeting predictor sites involving bioinformatics tools in databases (Fig. 1). Further the data regarding miRNAs cross interference was performed in neonatal cultured cardiomyocytes by employing microRNA inhibitors and later by expression analysis of miRNAs and respective target genes.

Data involves miR-15a, miR-29a and miR-214 complementary sequence matching of targeting pro- apoptotic genes as PUMA, DRP1 and ARC (Fig. 1). Dynamin related protein 1 (DRP-1) get overexpressed as a result of cellular stress and apoptosis while playing important role in mitochondrial fission [2]. The p53 up-regulated modulator of apoptosis (PUMA) is involved in DRP1 accumulation in mitochondrial membrane [3]. Data presenting miR-214 seed matching with ARC which is an apoptosis inhibitory protein. Apoptosis repressor with caspase recruitment domain (ARC) attenuates mitochondrial membrane depolarization by directly binding with PUMA whenever highly expressed in cardiomyocytes [4].

**Fig. 2:**
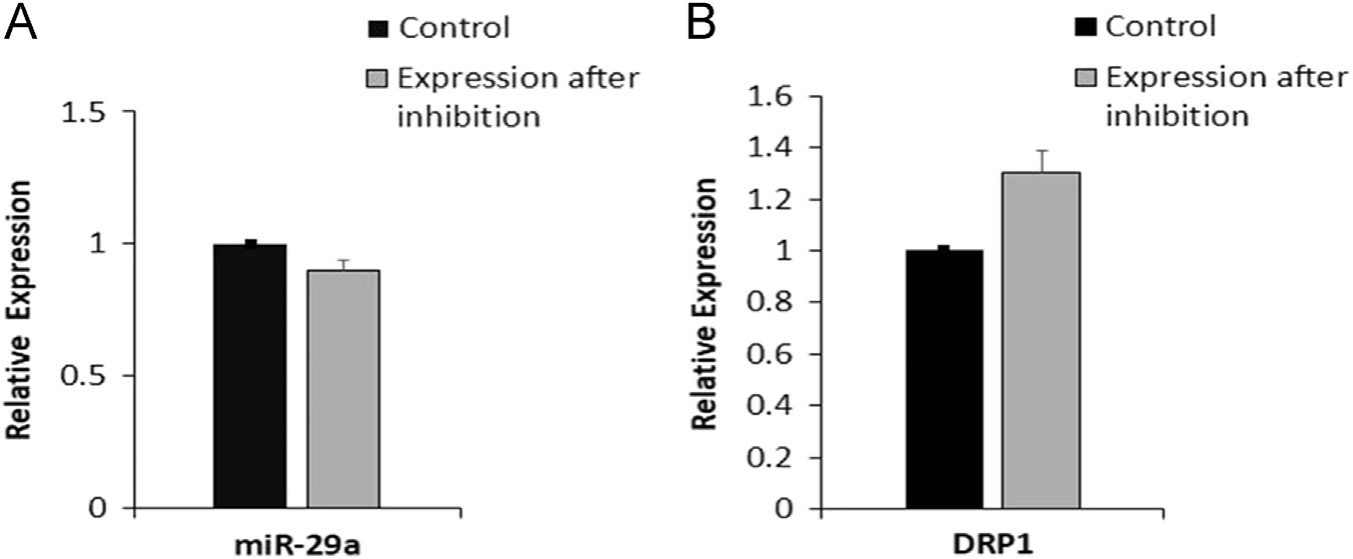
Expression of miR-29a and DRP1 in neonatal cardiomyocytes transfected with miRNA 15a inhibitor. miRNA and genes expression was performed by qRT-PCR. **A.** The relative expression of miR-29a after transfection with anti miR-15a. **B.** The relative expression level of DRP1 in inhibitor treated cardiomyocytes as compared to control.

**Fig. 3:**
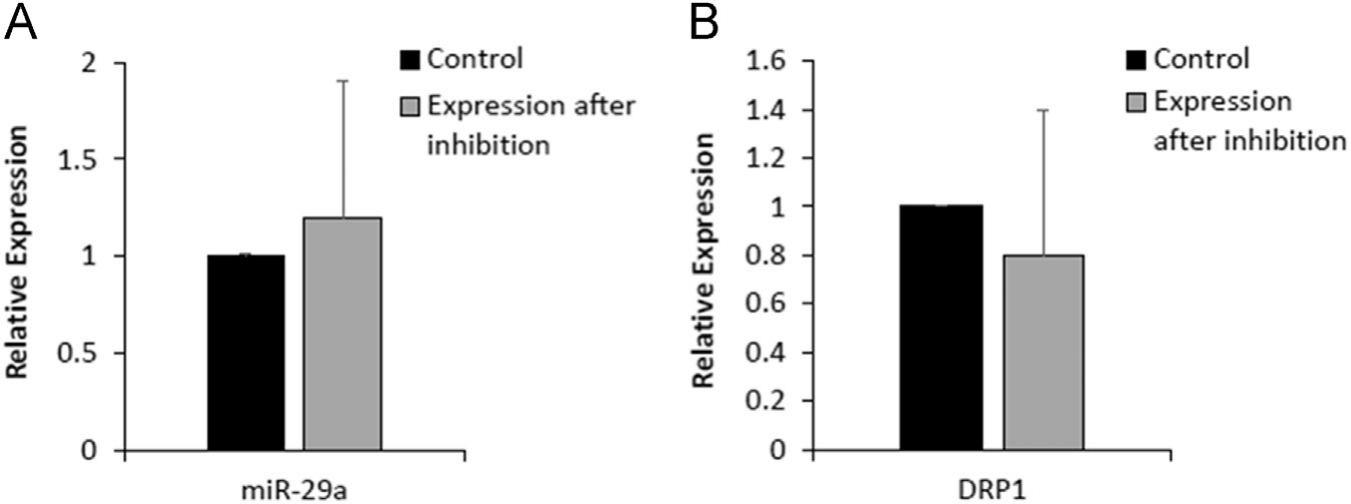
Expression of miR-29a and DRP1 in cultured neonatal cardiomyocytes after transfection with anti miR-214. **A.** The relative expression of miR-29a after employing mir-214 inhibitor. **B.** The relative expression of DRP1 after employing mir-214 inhibitor.

Data of miR-15a inhibitor treatment leading to reduction in miR-29a expression and subsequent increase in its target DRP1 expression to certain extent may point towards cross interference phenomenon in miRNA inhibition (Fig. 2). Further, the data shows opposite response in the case of miR-214 inhibitor treatment that leads to the minute up regulation of miR-29a and subsequent reduction in the expression of its target gene DRP1 (Fig. 3). The data presented here showed non significant alteration at expression level and this does not imply that specific miRNA inhibitor strongly attenuates the other miRNAs and respective targets. Our recent studies showed that miR-15a and miR-214 inhibitors treatment leads to significant inhibition of their target miRNAs and enhanced the expression of respective genes PUMA and ARC by many folds [1].

## 2 Experimental design, materials, and methods

### 2.1 Materials

0.05% trypsin − 0.02% EDTA in PBS (Invitrogen, USA), 0.1% trypsin − 0.02% EDTA in PBS (Invitrogen, USA), potassium phosphate buffer (pH 7) (Merck, USA), FCS (Gibco, USA), MEM, DMEM, Medium 199 (Gibco, USA), Gelatine 2% for TC (Sigma Aldrich, USA), miRNA inhibitors (IDT, Lowa, USA), SYBR Green qRT-PCR Kit (Thermo Fisher Scientific, USA).

### 2.2 miRNA target site prediction

miR-15a, miR-29 and miR-214 predicted targets were confirmed by multiple online databases as described [The TargetScan (http://www.targetscan.org), miRanda (www.miranda-im.org), PicTar (http://pictar.mdc-berlin.de), and miRBase (http://www.microrna.org)].

### 2.3 Culturing of cardiomyocytes

Neonatal Sprague Dawley rats were used for the current study and animal handling procedures were strictly under the guidelines and regulation of Bio-Ethics Committee of Quaid-i-Azam University Islamabad Pakistan. Neonatal rat's primary cardiomyocytes culturing was performed as described by Murtaza et al. [5]. Briefly, the cardiomyocytes were digested in 0.1% trypsin containing 0.02% EDTA in PBS and collagenase pancreatin mixture. After blocking the enzymatic activity, the harvested cells were re-suspended in complete medium containing 100 U/ml penicillin, 100 µg/ml streptomycin. Cardiomyocytes were separated from other cells of the heart tissues as fibroblasts and RBCs. The cardiomyocytes achieved in suspension were seeded on gelatin or polylysine coated plates. Total seeding density for cardiomyocytes was 1 × 10^6^ cells per well of 6 well plates.

### 2.4 miRNA inhibitors treatment

Cardiomyocytes were transfected with inhibitors of miR-15a and miR-214 (IDT Lowa, USA) by Calcium chloride transfection and Glycerol shock procedure as described by Mortensen et al. [6]. Total concentration of miRNA inhibitors was 10 nM and incubation time was 48 h.

### 2.5 Quantitative reverse transcription PCR

miRNAs and target genes expression was performed by quantitative real-time polymerase chain reaction (qRT-PCR) [7]. Briefly, total RNA was extracted with Trizol (Invitrogen, UK), and expression of target genes was quantified using a SYBR Green qRT-PCR Kit (Thermo Fisher Scientific, USA). qRT-PCR primers were from Thermo Fisher Scientific. For miRNAs, stem-loop qRT-PCR was performed [8]. Following DNase treatment, RNA was reverse transcribed. qPCR was performed by syber green master mix on the real Time PCR (Thermo fisher, USA). Gene expression was normalized to that of GAPDH or U6 for mRNAs and miRNAs, respectively. The expression of target genes relative to the control was determined using the 2^−ΔΔCT^ method [7].

### 2.6 Statistical analysis

Present data were expressed as mean value ± S.D by employing One-way analysis of variance (ANOVA) followed by the Tukey's. Data analysis was carried out by the SPSS (Chicago, IL, USA) 13.0 software. *P* value less than 0.05 was considered statistically significant.

## References

[1] Jan M.I., Khan R.A., Ali T., Bilal M., Bo L., Sajid A., Malik A., Urehman N., Waseem N., Nawab J., Ali M., Majeed A., Ahmad H., Aslam S., Hamera S., Sultan A., Anees M., Javed Q., Murtaza I. (2017). Interplay of mitochondria apoptosis regulatory factors and microRNAs in valvular heart disease. Arch. Biochem. Biophys..

[2] Frank S., Gaume B., Bergmann-Leitner E.S., Leitner W.W., Robert E.G., Catez F., Smith C.L., Youle R.J. (2001). The role of dynamin-related protein 1, a mediator of mitochondrial fission. Apoptosis Dev. Cell.

[3] Altin S.E., Schulze P.C. (2011). P53-upregulated modulator of apoptosis (PUMA): a novel proapoptotic molecule in the failing heart. Circulation.

[4] Ludwig-Galezowska A.H., Flanagan L., Rehm M. (2011). Apoptosis repressor with caspase recruitment domain, a multifunctional modulator of cell death. J. Cell. Mol. Med..

[5] Murtaza I., Wang H.X., Feng X., Alenina N., Bader M., Prabhakar B.S., Li P.F. (2008). Down-regulation of catalase and oxidative modification of protein kinase CK2 lead to the failure of apoptosis repressor with caspase recruitment domain to inhibit cardiomyocyte hypertrophy. J. Biol. Chem..

[6] Mortensen R.M., Kingston R.E. (2009). Selection of transfected mammalian cells. Curr. Protoc. Mol. Biol..

[7] Nigam V., Sievers H.H., Jensen B.C., Sier H.A., Simpson P.C., Srivastava D., Mohamed S.A. (2010). Altered microRNAs in bicuspid aortic valve: a comparison between stenotic and insufficient valves. J. Heart Valve Dis..

[8] Liu F., Li N., Long B., Fan Y.Y., Liu C.Y., Zhou Q.Y., Murtaza I., Wang K., Li P.F. (2014). Cardiac hypertrophy is negatively regulated by miR-541. Cell Death Dis..

